# Supplementary material for: The interactome of intact mitochondria by cross-linking mass spectrometry provides evidence for coexisting respiratory supercomplexes
Source: Mol Cell Proteomics. 2017 Dec 8;17(2):216–32. doi: 10.1074/mcp.RA117.000470 (PMC5795388; doi:10.1074/mcp.RA117.000470)
Supplement: Supplemental Data [file supp_RA117.000470_133922_0_supp_23978_fzffwf.zip › spectra_annotation/mito_DR_spectra_annotation/134-1-4-1-7-1.pdf]

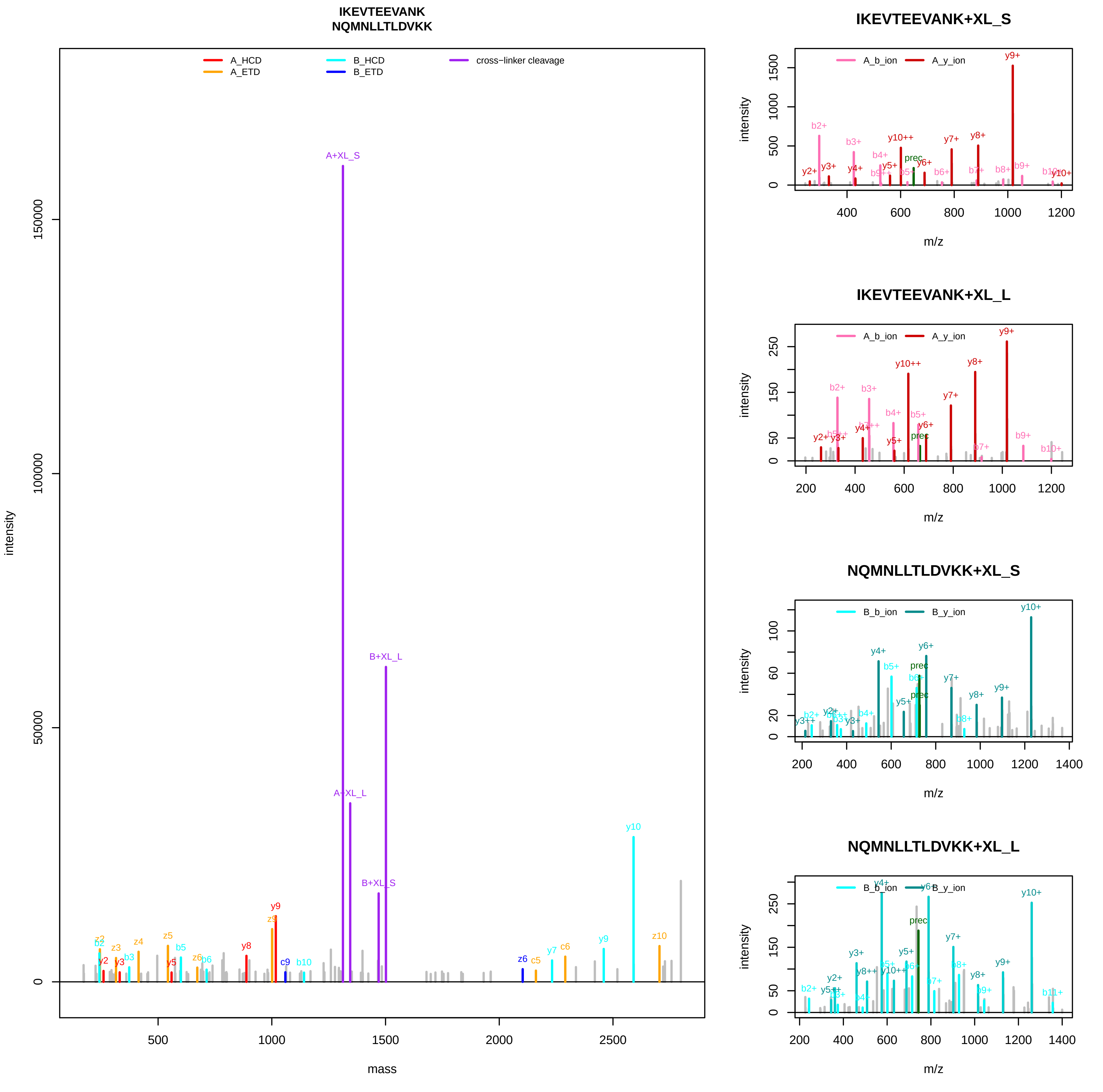
IKEVTEEVANK
NQMNLLTLDVKK
A_HCD
A_ETD
B_HCD
B_ETD
cross−linker cleavage
A+XL_S
B+XL_L
A+XL_L
B+XL_S
y10
z10
y9
z9
y8
c9
b10
z6
c5
y7
c6
z2
b2
y2
z3
y3
z4
b3
z5
y5
b5
z6
b6
intensity
mass
0
50000
100000
150000
500
1000
1500
2000
2500
IKEVTEEVANK+XL_S
A_b_ion
A_y_ion
y9+
b2+
b3+
y10++
y7+
y8+
b4+
prec
y6+
y5+
b9++
b5+
b6+
b7+
b8+
b9+
b10+
y10+
y2+
y3+
y4+
intensity
m/z
IKEVTEEVANK+XL_L
NQMNLLTLDVKK+XL_S
B_b_ion
B_y_ion
y10+
y4+
y6+
b5+
prec
y7+
y8+
y9+
y5+
y2+
b2+
y3++
y3+
b4+
b8+
NQMNLLTLDVKK+XL_L
b11+
